# Genome-Wide Identification and Analysis of Genes, Conserved between *japonica* and *indica* Rice Cultivars, that Respond to Low-Temperature Stress at the Vegetative Growth Stage

**DOI:** 10.3389/fpls.2017.01120

**Published:** 2017-06-30

**Authors:** Manu Kumar, Yun-Shil Gho, Ki-Hong Jung, Seong-Ryong Kim

**Affiliations:** ^1^Department of Life Sciences, Sogang UniversitySeoul, South Korea; ^2^Graduate School of Biotechnology and Crop Biotech Institute, Kyung Hee UniversityYongin, South Korea

**Keywords:** abiotic stress, cold stress, MapMan analysis, meta-expression analysis, Gene Ontology enrichment analysis, transcriptomics, rice, microarray

## Abstract

Cold stress is very detrimental to crop production. However, only a few genes in rice have been identified with known functions related to cold tolerance. To meet this agronomic challenge more effectively, researchers must take global approaches to select useful candidate genes and find the major regulatory factors. We used five Gene expression omnibus series data series of Affymetrix array data, produced with cold stress-treated samples from the NCBI Gene Expression Omnibus (http://www.ncbi.nlm.nih.gov/geo/), and identified 502 cold-inducible genes common to both *japonica* and *indica* rice cultivars. From them, we confirmed that the expression of two randomly chosen genes was increased by cold stress *in planta*. In addition, overexpression of *OsWRKY71* enhanced cold tolerance in ‘Dongjin,’ the tested *japonica* cultivar. Comparisons between *japonica* and *indica* rice, based on calculations of plant survival rates and chlorophyll fluorescence, confirmed that the *japonica* rice was more cold-tolerant. Gene Ontology enrichment analysis indicate that the ‘L-phenylalanine catabolic process,’ within the Biological Process category, was the most highly overrepresented under cold-stress conditions, implying its significance in that response in rice. MapMan analysis classified ‘Major Metabolic’ processes and ‘Regulatory Gene Modules’ as two other major determinants of the cold-stress response and suggested several key *cis*-regulatory elements. Based on these results, we proposed a model that includes a pathway for cold stress-responsive signaling. Results from our functional analysis of the main signal transduction and transcription regulation factors identified in that pathway will provide insight into novel regulatory metabolism(s), as well as a foundation by which we can develop crop plants with enhanced cold tolerance.

## Introduction

Agronomic productivity is declining due to various environmental problems, including cold stress. Crop yields are not sustainable when threatened by either chilling or freezing. The typical physiological response of a rice (*Oryza sativa*) plant exposed to such conditions is inhibited germination, followed by retarded seedling growth and restricted photosynthesis. Long periods of stress lead to chlorosis and tissue necrosis. Therefore, it is important that researchers improve their understanding of the regulatory mechanisms that can enhance cold tolerance.

The process of stress responses comprises perception of the low temperature, signal transduction, activation of TFs and stress-responsive genes, detoxification of reactive oxygen species (ROS), and initiation of repair systems. These steps make plants more tolerant to cold stress. Genetic and molecular studies have elucidated the functions of 59 such genes, for which information is now well-summarized in the OGRO database^1^ (Yamamoto et al., 2012). Many important crops, including rice, are sensitive to low temperatures and do not easily acclimatize during periods of cold stress. At the seedling stage, rice is more vulnerable, even to mild chilling. This can reduce overall growth and disrupt and delay the cycle of crop maturation, eventually decreasing yields (Zhang et al., 2014). The challenge of global warming means that crop plants, including rice, will be more exposed to extreme growing environments, e.g., low and high temperatures. Although the response by rice to cold stress has been described (Zhi-guo et al., 2014; Wang D. et al., 2016; Shakiba et al., 2017), we still need to identify more effective genes that can regulate this response.

Transcriptome analysis is a very powerful tool that provides the global view of a phenomenon and frequently suggests novel candidate genes for further study. Such analyses have been conducted to improve our understanding about the cold-stress response in rice. For example, (Zhang T. et al., 2012) have found more than 500 candidate genes that are significantly up-regulated under low temperatures. Moreover, 183 DEGs related to cold stress have been identified by Chawade et al. (2013), 383 DEGs by Yang et al. (2015), and more than 2000 DEGs by Zhao et al. (2014). Nevertheless, it has been difficult to determine from publicly available transcriptome data which of these candidate genes show consistent expression patterns under stress as well as across a range of cultivars.

Here, we focused on genes that are consistently up-regulated between *japonica* and *indica* cultivars under cold stress at the seedling stage. Our investigation utilized a large set of transcriptome data consisting of 27 *japonica* and 36 *indica* comparisons under low-temperature conditions, as obtained from the NCBI GEO (Barrett et al., 2011). From this, we identified 502 candidate genes that we further analyzed for their biological significance using GO term enrichment analysis and functional classifications via MapMan analysis^2^. We also selected two genes and confirmed their cold-inducible expression patterns using promoter-GUS trap systems. Based on those results, we proposed a novel promoter for further research applications to enhance cold tolerance. We then developed a hypothetical model to describe the signaling and transcriptional regulatory pathways that process the response to cold stress in rice.

## Materials and Methods

### Plant Materials and Stress Treatments

Plants of *japonica* rice cv. Dongjin (‘DJ’) and *indica* rice ‘IR64’ (‘IR64’) were grown in a walk-in chamber (Koencon, Hanam, South Korea) under conditions of 30°C [200 μmol m^-2^ s^-1^ (day)]/22°C (night) and a 12-h photoperiod for 10 days in plastic boxes containing 100 g of soil used in growing rice (Punong, Kyung-Ju, Korea) (Kumar et al., 2017). The effects of cold stress (exposure at 4°C) on the light intensity 110 μmol m^-2^ s^-1^ were examined after exposure to cold stress for 0, 24 h/1 day, 48 h/2 days, 72 h/3 days, 96 h/4 days, 120 h/5 days, and 144 h/6 days using chlorophyll fluorescence. Our mock treatment comprised a group of plants that remained at the normal growing temperature (28°C) throughout the experimental period. To observe the physiological features of these seedlings, we used samples collected before cold stress was induced, as well as from plants after 4 days of stress, and then after recovery under normal conditions for 5 days. Fresh weights (FWs) were recorded after recovery from cold stress, and dry weights (DWs) were measured after the samples were dried at 80°C for 2 days.

### RT and qRT-PCR Analysis

For monitoring the expression of cold-inducible marker genes, seedlings (selected at 10 DAG, or 10 DAG) were hydroponically cultured in Yoshida solution and exposed to 4°C for 0, 1, 3, 6, 12, or 24 h. Primers of *OsZFP182/LOC_Os03g60560* and *OsWYRKY71/LOC_Os02g08440* were used for RT and qRT-PCR analyses at a final concentration of 10 pmol, with 3 μL (equivalent to 30 ng of total RNA) of cDNA as template (Supplementary Table S1). The internal controls were primers of rice *ubiquitin 5* (Os*Ubi5*) and rice *actin 1* (*RAc1*) (Supplementary Table S1). An RNeasy Mini Plant Kit (Qiagen, Germany) was used for total RNA isolation and an RT Complete Kit (Biofact, Korea) was used for cDNA synthesis according to the manufacturers’ instructions. Primers were designed with Gene Runner software^3^ and NCBI primer blast^4^. The amplified products were resolved on a 1% agarose gel.

### Measurement of H_2_O_2_

An uptake assay was conducted to determine the relative concentration of H_2_O_2_, using Amplex^®^ Red reagent (10-acetyle-3, 7dihydroxyphenoxazine; Molecular Probes/Invitrogen, United States) (Mohanty et al., 1997). Leaf tissues (0.1 mg μL^-1^) were homogenized in a standard MS medium (Murashige and Skoog, 1962) and then incubated under darkness for 30 min with horseradish peroxidase (0.2 U mL^-1^) and Amplex^®^ Red reagent (1 μM). The H_2_O_2_ released from these tissues was detected by a SpectraMax 250 Microplate Reader (Molecular Devices Inc., United States) with absorbance measured at 560 nm (Kumar et al., 2014).

### Meta-Expression Analysis

We downloaded raw data for five GSE data series (i.e., GEO accession number GSE6901, GSE33204, GSE37940, GSE38023, and GSE31077) that are related to cold-stress responses, as indicated from the NCBI GEO^5^ (Barrett et al., 2011). Details are presented in Supplementary Table S2. The data were normalized using an Affy Package encoded by R language, and the intensity values were transformed into the log_2_ scale as we have previously described (Cao et al., 2012). This allowed us to generate log_2_ fold-change values for cold-stressed samples. Similar fold-changes were revealed for other stress conditions. For each data series, we used those fold-change data to perform a KMC analysis to identify genes that were consistently up-regulated under all cold-stress conditions. The KMC analysis of meta-expression data for abiotic stresses – salt, drought, cold, heat, submergence, and anaerobic conditions – grouped all of the candidate genes into 12 clusters. From these, we selected 502 genes that were up-regulated by cold-stress treatment but not during the recovery period. Heatmap images were produced using Mev software (Chu et al., 2008).

### *GUS* Assays and Co-segregation Test of Promoter Trap Lines

To examine *GUS* expression patterns, we germinated seeds from two promoter trap lines in an MS medium for 7 days. These lines were obtained from a mixed pool of PFG T-DNA tagging lines from POSTECH in Korea (Lee et al., 2004; Jung et al., 2005, 2006, 2015; Hong et al., 2017; Wei et al., 2017). The resultant plantlets were then exposed to cold stress (4°C) for 0 or 24 h. Afterward, whole seedlings from all treatment groups were soaked for 8 h in a *GUS*-staining solution before their roots were photographed with a camera (Canon EOS 550D; Cannon, Tokyo, Japan).

### Analysis of *Cis*-Regulatory Elements

To identify any consensus CREs in the promoters of our cold-inducible genes, we extracted 2-kb upstream sequences of ATG for *LOC_Os01g31370* and *LOC_Os03g49830*, which were validated in our current *GUS* assays. We also used the sequence for *LOC_Os10g41200*, which was previously reported to be a cold-inducible promoter based on the promoter-*GUS* system (Rerksiri et al., 2013; Jeong and Jung, 2015) from PLANTPAN^6^ (Chang et al., 2008). Several MEME searches were performed with those sequences in the FASTA format via the Web server hosted by the National Biomedical Computation Resource^7^. We looked for up to five CREs with an option of 12 maximum motif widths. Using the MAST, we then searched DNA sequences for matches to the putative TOMTOM within a set of promoter sequences (Bailey et al., 2006).

### Analysis of Gene Ontology Enrichment

To analyze the biological significance of selected candidate genes, we employed the GO enrichment tool installed in the Rice Oligonucleotide Array Database^8^ (Jung et al., 2008a; Cao et al., 2012). For this, we uploaded 502 genes showing upregulation in both *japonica* and *indica* cultivars under cold stress. A fold-enrichment value higher than the standard (‘1’) meant that the selected GO term was over-represented more than was expected. Terms with >2-fold enrichment values and *p*-values < 0.05 were also used as criteria for choosing the most significant GO terms in the ‘Biological Process’ category.

### MapMan Analysis

The rice MapMan classification system covers 36 BINs, each of which can be extended in a hierarchical manner into subBINs (Usadel et al., 2005; Urbanczyk-Wochniak et al., 2006). By applying diverse MapMan tools, a significant gene list selected from high-throughput data analysis can be integrated to diverse overviews. Here, we uploaded locus IDs from the RGAP for 502 DEGs with a value of ‘3,’ which indicated upregulation under cold stress. Finally, we used four overviews – Metabolism, Regulation, Transcription, and Proteasome – installed in the MapMan toolkit.

### Analysis of Rice Genes with Known Functions

To evaluate the functional significance of our candidate genes, we compared our list with the one from OGRO, which summarizes rice genes with known functions (**Table 1**; Yamamoto et al., 2012).

**Table 1:** Rice genes functionally characterized as cold-inducible.

Gene	Major_F	Minor_F	RAP-DB_ID	MSU_ID	Method	Detailed functions	Reference
*OsDREB1C*	R/T	Cold T	*Os06g0127100*	*LOC_Os06g03670.1*	OX	Cold, drought, and salinity T.	Ito et al., 2006
*ZFP182*	R/T	Cold T	*Os03g0820300*	*LOC_Os03g60560.1*	OX	Cold, drought, and salinity T.	Huang et al., 2012
*OsDREB1B*	R/T	Cold T	*Os09g0522000*	*LOC_Os09g35010.1*	OX	Cold, drought, and salinity T.	Ito et al., 2006
*OsDREB1A*	R/T	Cold T	*Os09g0522200*	*LOC_Os09g35030.1*	OX	Cold, drought, and salinity T.	Ito et al., 2006
*OsWRKY45*	R/T	Cold T	*Os05g0322900*	*LOC_Os05g25770.1*	Kd OX	Cold, drought, and salinity T; ABA sensitivity.	Tao et al., 2011
*OsWRKY71*	R/T	Cold T	*Os02g0181300*	*LOC_Os02g08440.1*	OX	Cold T	Kim et al., 2016
*OsTPP1*	R/T	Cold T	*Os02g0661100*	*LOC_Os02g44230.1*	OX	Cold and salinity T.	Ge et al., 2008
*OsWRKY76*	R/T	Cold T	*Os09g0417600*	*LOC_Os09g25060.1*	OX	R to *Magnaporthe oryzae*; cold T.	Yokotani et al., 2013
*OsMYB2*	R/T	Cold T	*Os03g0315400*	*LOC_Os03g20090.1*	OX	Cold, drought, and salinity T; ABA sensitivity.	Yang et al., 2012
*OsCAF1B*	R/T	Cold T	*Os04g0684900*	*LOC_Os04g58810.1*	Others	Cold T	Chou et al., 2014
*OsMAPK5*	R/T	Cold T	*Os03g0285800*	*LOC_Os03g17700.1*	OX	R to *Magnaporthe grisea* and *Burkholderia glumae*; cold, drought, and salinity T.	Xiong and Yang, 2003
*OsbZIP52/RISBZ5*	R/T	Cold T	*Os06g0662200*	*LOC_Os06g45140.1*	OX	Cold and drought T.	Liu et al., 2012
*OsSPX1*	R/T	Cold T	*Os06g0603600*	*LOC_Os06g40120.1*	Kd	Cold and oxidative stresses T.	Wang C. et al., 2013
*OsDREB1C*	R/T	Drought T	*Os06g0127100*	*LOC_Os06g03670.1*	OX	Cold, drought, and salinity T.	Ito et al., 2006
*ZFP182*	R/T	Drought T	*Os03g0820300*	*LOC_Os03g60560.1*	OX	Cold, drought, and salinity T.	Huang et al., 2012
*OsSRO1c*	R/T	Drought T	*Os03g0230300*	*LOC_Os03g12820.1*	Mutant	Stomatal control; oxidative stress R.	You et al., 2013
*OsDREB1B*	R/T	Drought T	*Os09g0522000*	*LOC_Os09g35010.1*	OX	Cold, drought, and salinity T.	Ito et al., 2006
*OsDREB1A*	R/T	Drought T	*Os09g0522200*	*LOC_Os09g35030.1*	OX	Cold, drought, and salinity T.	Ito et al., 2006
*OsWRKY45*	R/T	Drought T	*Os05g0322900*	*LOC_Os05g25770.1*	Kd OX	Cold, drought, and salinity T; ABA sensitivity.	Tao et al., 2011
*OsMYB2*	R/T	Drought T	*Os03g0315400*	*LOC_Os03g20090.1*	OX	Cold, drought, and salinity T; ABA sensitivity.	Yang et al., 2012
*OsbHLH148*	R/T	Drought T	*Os03g0741100*	*LOC_Os03g53020.1*	OX	Drought T.	Seo et al., 2011
*OsCAF1B*	R/T	Drought T	*Os04g0684900*	*LOC_Os04g58810.1*	Others	Drought T	Chou et al., 2014
*OsMAPK5*	R/T	Drought T	*Os03g0285800*	*LOC_Os03g17700.1*	Kd OX	R to *Magnaporthe grisea* and *Burkholderia glumae*; cold, drought, and salinity T.	Xiong and Yang, 2003
*OsbZIP52/RISBZ5*	R/T	Drought T	*Os06g0662200*	*LOC_Os06g45140.1*	OX	Cold and drought T.	Liu et al., 2012
*ONAC045*	R/T	Drought T	*Os11g0127600*	*LOC_Os11g03370.1*	OX	Drought and salinity T.	Zheng et al., 2009
*OsCDPK7*	R/T	Drought T	*Os04g0584600*	*LOC_Os04g49510.1*	OX	Drought and salinity T.	Saijo et al., 2000
*OsCPK4*	R/T	Drought T	*Os02g0126400*	*LOC_Os02g03410.1*	Kd	Protection of cellular membrane from drought stress.	Campo et al., 2014
*OsERF3*	R/T	Drought T	*Os01g0797600*	*LOC_Os01g58420.1*	OX	Drought T by controlling ethylene biosynthesis.	Wan et al., 2011
*OsAP2-39*	R/T	Drought T	*Os04g0610400*	*LOC_Os04g52090.1*	OX	Dwarfism; fertility; and drought T.	Yaish et al., 2010
*OsDREB1C*	R/T	Salinity T	*Os06g0127100*	*LOC_Os06g03670.1*	OX	Cold, drought, and salinity T.	Ito et al., 2006
*OsEATB*	R/T	Salinity T	*Os09g0457900*	*LOC_Os09g28440.1*	OX	Internode elongation; panicle branching; tillering; salinity T.	Qi et al., 2011
*ZFP182*	R/T	Salinity T	*Os03g0820300*	*LOC_Os03g60560.1*	OX	Cold, drought, and salinity T.	Huang et al., 2012
*ZFP179*	R/T	Salinity T	*Os01g0839100*	*LOC_Os01g62190.1*	OX	Salinity and oxidative stress T.	Sun et al., 2010
*OsDREB1B*	R/T	Salinity T	*Os09g0522000*	*LOC_Os09g35010.1*	OX	Cold, drought, and salinity T.	Ito et al., 2006
*OsDREB1A*	R/T	Salinity T	*Os09g0522200*	*LOC_Os09g35030.1*	OX	Cold, drought, and salinity T.	Ito et al., 2006
*OsWRKY45*	R/T	Salinity T	*Os05g0322900*	*LOC_Os05g25770.1*	Kd OX	Cold, drought, and salinity T; ABA sensitivity.	Tao et al., 2011
*OsTPP1*	R/T	Salinity T	*Os02g0661100*	*LOC_Os02g44230.1*	OX	Cold and salinity T.	Ge et al., 2008
*OsMYB2*	R/T	Salinity T	*Os03g0315400*	*LOC_Os03g20090.1*	OX	Cold, drought, and salinity T; ABA sensitivity.	Yang et al., 2012
*OsMAPK5*	R/T	Salinity T	*Os03g0285800*	*LOC_Os03g17700.1*	Kd OX	R to *Magnaporthe grisea* and *Burkholderia glumae*; cold, drought, and salinity T.	Xiong and Yang, 2003
*ONAC045*	R/T	Salinity T	*Os11g0127600*	*LOC_Os11g03370.1*	OX	Drought and salinity T.	Zheng et al., 2009
*OsCDPK7*	R/T	Salinity T	*Os04g0584600*	*LOC_Os04g49510.1*	OX	Drought and salinity T.	Saijo et al., 2000
*OsCPK4*	R/T	Salinity T	*Os02g0126400*	*LOC_Os02g03410.1*	Kd	Protection of cellular membrane from salt stress.	Campo et al., 2014
							


*OsPLDbeta1*	R/T	Blast R	*Os10g0524400*	*LOC_Os10g38060.1*	Kd	R to *Pyricularia grisea* and *Xanthomonas oryzae* pv. *oryzae*.	Yamaguchi et al., 2009
*OsWRKY45*	R/T	Blast R	*Os05g0322900*	*LOC_Os05g25770.1*	Kd OX	R to *Xanthomonas oryzae* pv. *oryzae*, pv. *oryzicola*, and *Magnaporthe grisea*.	Tao et al., 2011
*OsAOS2*	R/T	Blast R	*Os03g0225900*	*LOC_Os03g12500.1*	OX	R to *Magnaporthe grisea*.	Mei et al., 2006
*OsWRKY76*	R/T	Blast R	*Os09g0417600*	*LOC_Os09g25060.1*	OX	R to *Magnaporthe oryzae*; cold T.	Yokotani et al., 2013
*OsMAPK5*	R/T	Blast R	*Os03g0285800*	*LOC_Os03g17700.1*	Kd OX	R to *Magnaporthe grisea* and *Burkholderia glumae*; cold, drought, and salinity T.	Xiong and Yang, 2003
*OsbHLH65*	R/T	Blast R	*Os04g0493100*	*LOC_Os04g41570.1*	Others	Defense R against rice blast.	Shin et al., 2014
*OsWRKY45*	R/T	Bacterial blight R	*Os05g0322900*	*LOC_Os05g25770.1*	Kd OX	R to *Xanthomonas oryzae* pv. *oryzae*, pv. *oryzicola*, and *Magnaporthe grisea*.	Tao et al., 2011
*OsWRKY76*	R/T	Bacterial blight R	*Os09g0417600*	*LOC_Os09g25060.1*	OX	R to *Xanthomonas oryzae* pv. *oryzae*.	Yokotani et al., 2013
*OsMAPK5*	R/T	Bacterial blight R	*Os03g0285800*	*LOC_Os03g17700.1*	Kd OX	R to *Magnaporthe grisea* and *Burkholderia glumae*; cold, drought, and salinity T.	Xiong and Yang, 2003
*OsNAC4*	R/T	Bacterial blight R	*Os01g0816100*	*LOC_Os01g60020.1*	Kd	Bacterial blight R; HR cell death.	Kaneda et al., 2009
*OsWRKY71*	R/T	Bacterial blight R	*Os02g0181300*	*LOC_Os02g08440.1*	OX	R to *Xanthomonas oryzae* pv. *oryzae*.	Liu et al., 2007
*OsHI-LOX*	R/T	Insect R	*Os08g0508800*	*LOC_Os08g39840.1*	Kd	R to rice striped stem borer and rice brown planthopper.	Zhou et al., 2009
*OsWRKY45*	R/T	Sheath blight R	*Os05g0322900*	*LOC_Os05g25770.1*	Kd OX	R to *Xanthomonas oryzae*, *Magnaporthe grisea* and *Rhizoctonia solani*.	Tao et al., 2011
							
*OsMAPK5*	R/T	Other disease R	*Os03g0285800*	*LOC_Os03g17700.1*	Kd OX	R to *Magnaporthe grisea* and *Burkholderia glumae*; cold, drought, and salinity T.	Xiong and Yang, 2003
*OsSRO1c*	R/T	Other stress R	*Os03g0230300*	*LOC_Os03g12820.1*	Mutant	Apoplastic and chloroplastic oxidative stress T; temperature stress T.	You et al., 2013
*OsCAF1B*	R/T	Other stress R	*Os04g0684900*	*LOC_Os04g58810.1*	Others	Wounding; ABA T.	Chou et al., 2014
*OsSPX1*	R/T	Other stress R	*Os06g0603600*	*LOC_Os06g40120.1*	Kd	Cold and oxidative stress T.	Wang C. et al., 2013
*ZFP179*	R/T	Other soil stress T	*Os01g0839100*	*LOC_Os01g62190.1*	OX	Salinity and oxidative stress T.	Sun et al., 2010
*OsSPX1*	R/T	Other soil stress T	*Os06g0603600*	*LOC_Os06g40120.1*	OX	Phosphate homeostasis.	Wang C. et al., 2013
*OsEATB*	MT	Dwarf	*Os09g0457900*	*LOC_Os09g28440.1*	OX	Internode elongation; panicle branching; tillering; salinity T.	Qi et al., 2011
*OsPHI-1*	MT	Dwarf	*Os02g0757100*	*LOC_Os02g52040.1*	Kd	Dwarfism.	Aya et al., 2014
*OsMPS*	MT	Dwarf	*Os02g0618400*	*LOC_Os02g40530.1*	Kd OX	Grain size; total biomass.	Schmidt et al., 2013
*RERJ1*	MT	Dwarf	*Os04g0301500*	*LOC_Os04g23550.1*	Kd OX	Dwarfism; JA sensitivity during seedling stage.	Kiribuchi et al., 2004
*GA2ox3*	MT	Dwarf	*Os01g0757200*	*LOC_Os01g55240.1*	Others	Dwarfism; gibberellin catabolism.	Lo et al., 2008
*TIFY11b*	MT	Dwarf	*Os03g0181100*	*LOC_Os03g08330.1*	OX	Grain size; plant height.	Hakata et al., 2012
*OsDOG*	MT	Dwarf	*Os08g0504700*	*LOC_Os08g39450.1*	OX	Dwarfism; cell elongation; regulation of gibberellin biosynthesis.	Liu et al., 2011
*OsBZR1*	MT	Dwarf	*Os07g0580500*	*LOC_Os07g39220.1*	Kd	Dwarfism; leaf angle; brassinosteroid sensitivity.	Bai et al., 2007
*gid1*	MT	Dwarf	*Os05g0407500*	*LOC_Os05g33730.1*	Mutant	Dwarfism; gibberellin sensitivity.	Ueguchi-Tanaka et al., 2005
*cZOGT1*	MT	Dwarf	*Os04g0556500*	*LOC_Os04g46980.1*	OX	Dwarfism; leaf senescence; crown root.	Kudo et al., 2012
*brd1*	MT	Dwarf	*Os03g0602300*	*LOC_Os03g40540.1*	Mutant	Dwarfism; brassinosteroid biosynthesis.	Mori et al., 2002
*OsCPK4*	MT	Dwarf	*Os02g0126400*	*LOC_Os02g03410.1*	Kd	Dwarfism.	Campo et al., 2014
*OsAP2-39*	MT	Dwarf	*Os04g0610400*	*LOC_Os04g52090.1*	OX	Dwarfism; fertility; drought T.	Yaish et al., 2010
*RERJ1*	MT	Shoot seedling	*Os04g0301500*	*LOC_Os04g23550.1*	Kd OX	Dwarfism; JA sensitivity during seedling stage.	Kiribuchi et al., 2004
*CYP85A1*	MT	Shoot seedling	*Os03g0602300*	*LOC_Os03g40540.1*	Others	Rice lamina bending and leaf unrolling by promoting castasterone (CS).	Asahina et al., 2014
*kch1*	MT	Shoot seedling	*Os12g0547500*	*LOC_Os12g36100.1*	Mutant	Coleoptile elongation.	Frey et al., 2010
*OsWRKY42*	MT	Culm leaf	*Os02g0462800*	*LOC_Os02g26430.1*	OX	Promotion of leaf senescence through ROS accumulation; plant death.	Han et al., 2014
*OsEATB*	MT	Culm leaf	*Os09g0457900*	*LOC_Os09g28440.1*	OX	Internode elongation; panicle branching; tillering; salinity T.	Qi et al., 2011
*OsPHI-1*	MT	Culm leaf	*Os02g0757100*	*LOC_Os02g52040.1*	Kd	Cell size and number in culm (increased number of smaller parenchyma cells)	Aya et al., 2014
*OsBZR1*	MT	Culm leaf	*Os07g0580500*	*LOC_Os07g39220.1*	Kd	Dwarfism; leaf angle; brassinosteroid sensitivity.	Bai et al., 2007
*OsIAA23*	MT	Root	*Os06g0597000*	*LOC_Os06g39590.1*	Mutant	Root development; quiescent center identity; auxin sensitivity.	Ni et al., 2011
*MAIF1*	MT	Root	*Os02g0671100*	*LOC_Os02g44990.1*	OX	Seed germination; ABA sensitivity; root growth.	Yan et al., 2011
*EL5*	MT	Root	*Os02g0559800*	*LOC_Os02g35329.1*	Others	Maintenance of cell viability of root primordia.	Koiwai et al., 2007
*cZOGT1*	MT	Root	*Os04g0556500*	*LOC_Os04g46980.1*	OX	Dwarfism; leaf senescence; crown root.	Kudo et al., 2012
*OsCPK4*	MT	Root	*Os02g0126400*	*LOC_Os02g03410.1*	OX	Regulation of Na+ accumulation.	Campo et al., 2014
*Rdd1*	MT	Seed	*Os01g0264000*	*LOC_Os01g15900.1*	Kd OX	Grain length and width; 1000-grain weight; flowering time.	Iwamoto et al., 2009
*OsMPS*	MT	Seed	*Os02g0618400*	*LOC_Os02g40530.1*	Kd OX	Grain size; total biomass.	Schmidt et al., 2013
*TIFY11b*	MT	Seed	*Os03g0181100*	*LOC_Os03g08330.1*	OX	Grain size; plant height.	Hakata et al., 2012
*OsEATB*	MT	Panicle flower	*Os09g0457900*	*LOC_Os09g28440.1*	OX	Internode elongation; panicle branching; tillering; salinity T.	Qi et al., 2011
*MSF1*	MT	Panicle flower	*Os05g0497200*	*LOC_Os05g41760.1*	Mutant	Spikelet determinacy; floral organ development.	Ren et al., 2016
*OsAP2-39*	PT	Sterility	*Os04g0610400*	*LOC_Os04g52090.1*	OX	Dwarfism; fertility; drought T.	Yaish et al., 2010
*OsCHR4*	PT	Source activity	*Os07g0497000*	*LOC_Os07g31450.1*	Mutant	Chloroplast development in adaxial mesophyll.	Zhao et al., 2012
*BE1*	PT	Source activity	*Os06g0726400*	*LOC_Os06g51084.1*	Others	Starch granule-binding, amylopectin structure.	Abe et al., 2014
*MAIF1*	PT	Germination dormancy	*Os02g0671100*	*LOC_Os02g44990.1*	OX	Seed germination; ABA sensitivity; root growth.	Yan et al., 2011
*PLDβ1*	PT	Germination dormancy	*Os10g0524400*	*LOC_Os10g38060.1*	Kd	Sensitivity to ABA during germination stage.	Li and Xue, 2007
*Rdd1*	PT	Flowering	*Os01g0264000*	*LOC_Os01g15900.1*	Kd OX	Grain length and width; 1000-grain weight; flowering time.	Iwamoto et al., 2009
*etr2*	PT	Flowering	*Os04g0169100*	*LOC_Os04g08740.1*	Mutant	Flowering time; ethylene sensitivity; stem starch content.	Wuriyanghan et al., 2009
*SPK1(SYG1)*	PT	Others	*Os06g0603600*	*LOC_Os06g40120.1*	Kd OX	Pi-dependent inhibitor of Phosphate starvation response regulator 2 (PHR2).	Wang et al., 2014
*AFT*	Others	Others	*Os01g0185300*	*LOC_Os01g09010.1*	Kd	Ester-linked ferulate content in cell walls.	Piston et al., 2009
*etr2*	Others	Others	*Os04g0169100*	*LOC_Os04g08740.1*	Mutant	Flowering time; ethylene sensitivity; stem starch content.	Wuriyanghan et al., 2009
*OsExo1*	Others	Others	*Os01g0777300*	*LOC_Os01g56940.1*	OX	Processing of double-strand break sites.	Kwon et al., 2012

*R, resistant; T, tolerant; MT, morphological trait; OX, overexpression; Kd, knockdown*.

### Evaluation of Cold Tolerance in a Line Over-Expressing *OsWYRKY71*

Plants from an Ox line for *OsWYRKY71* (*OsWYRKY71*-Ox) under the control of CaMV35S promoter (Kim et al., 2016) and from the WT (*Japonica* cv. *Dongjin*) were grown for 10 days in plastic boxes containing soil. To test their tolerance, we then exposed them to cold stress (4^o^C) for 5 days and then returned them to normal growing conditions for 6 days of recovery. Survival rates were determined at the end of this experimental period. Cold stress analysis of *OsWYRKY71*-Ox lines was done with three replicates.

## Results and Discussion

### Physiological Responses of Cold-Stressed Rice Seedlings

**FIGURE 1:**
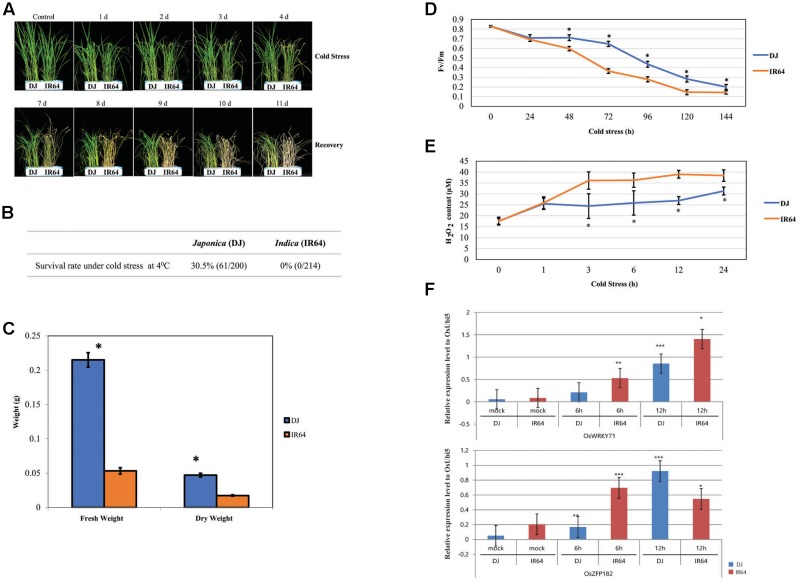
Analysis of cold stress responses by *japonica* and *indica* rice cultivars. **(A)** Phenotypes associated with cold-stress response by ‘DJ’ and ‘IR64’ rice seedlings observed during treatment for 4 days followed by 5 days of recovery. **(B)** Tolerance of seedlings based on survival rates. **(C)** Fresh and dry weights after recovery from cold stress. **(D)** Photosynthetic efficiency (*F*_v_/*F*_m_) after cold treatment for 4 days. **(E)** Determination of ROS concentrations (i.e., levels of H_2_O_2_) in seedlings after cold treatment for 24 h. **(F)** Expression of 2 marker genes (*OsZFP182* and *OsWRKY71*) in stressed seedlings, using *OsUbi5* as an internal control. ^∗∗∗^, *p*-value < 0.001, ^∗∗^, 0.001< *p*-value < 0.01; ^∗^, 0.01 < *p*-value < 0.05.

Cold stress adversely affects plant growth and yield, and rice isparticularly susceptible at the seedling stage (Zhang et al., 2014). Ouranalysis involved 10-day-old ‘DJ’ (*japonica*) and ‘IR64’(*indica*) plants exposed to 4°C for 4 days.Afterward, they recovered for 5 days at 28°C. Their phenotypes are shown in **Figure 1A**. At the end of this experimental period, the survival rate was 30.5% for ‘DJ’ versus 0.0% for ‘IR64,’indicating that the former was more old-tolerant (**Figure 1B**). The *F*_W_ value was 162 mg higher for ‘DJ’ while its DW was 29 mg higher than for ‘IR64’ (**Figure 1C**). Prolonged cold stress also negatively affected photosynthetic efficiency, with both cultivars showing significant reductions after 24 h (**Figure 1D**). The decline in efficiency after 48 h was more severe for ‘IR64’ than for ‘DJ.’

The accumulation of ROS, including H_2_O_2_, is a major indicator of the plant response to various abiotic stresses. We found that ‘IR64’ had higher H_2_O_2_ concentrations than did ‘DJ’ after 3 and 24 h of cold treatment (**Figure 1E**).

We also evaluated the expression patterns of two well-known cold stress-responsible genes, *OsZFP182* and *OsWRKY71* (Huang et al., 2007; Kim et al., 2016) and found that, as expected, their expression was significantly induced, and to nearly the same extent, in both cultivars (**Figure 1F**). This demonstrated that the tool of global transcriptome data can be broadly applied for determining and, ultimately, improving cold tolerance in rice.

### Genome-Wide Identification of Cold Stress-Inducible Genes in Both *japonica* and *indica* Cultivars Using Meta-Expression Data Analysis

**FIGURE 2:**
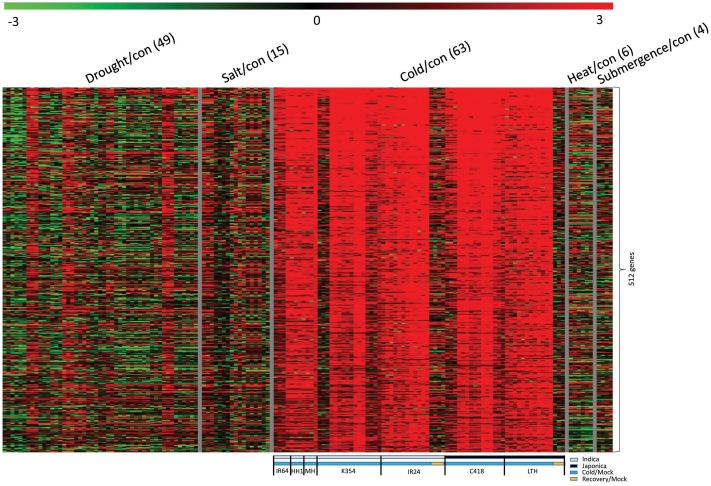
Heatmap of genes up-regulated under stress in both *japonica* and *indica* cultivars. Panel above heatmap indicates type of abiotic stress applied; parentheses indicate number of stress/control in each treatment. Panel below heatmap shows detailed information for “main target” samples under cold stress. Gray box, *indica* cultivars; black, *japonica* cultivars; blue, cold stress/control; and brown, recovery/control. *Indica* cultivars: ‘IR64,’ ‘Huahui 1’ (‘HH1’), ‘Minghui 63’ (‘MH’), ‘K354,’ and ‘IR24’; *japonica* cultivars: ‘C418’ and ‘Li-Jiang-Xin-Tuan-Hei-Gu’ (‘LTH’).

As a quantitative trait, tolerance to cold stress is governed by different sets of genes, and through diverse mechanisms. We used meta-expression analysis with transcriptome data and downloaded information about global candidate genes from the NCBI GEO for series GSE37940 and GSE38023 (Zhang F. et al., 2012; Zhang H. et al., 2012). After normalizing these data, we generated 63 comparisons for cold-stress treatment, as well as 49 comparisons for drought stress, 6 for high temperatures, and 4 for submergence (Supplementary Table S2). Our KMC analysis with the resultant fold-change data revealed 502 genes that were significant up-regulated upon cold stress but not under recovery conditions (**Figure 2**). From this, we prepared 27 comparisons with two *japonica* cultivars – ‘C418’ (a *japonica* restorer line for hybrid rice production and cold sensitive) and ‘Li-Jiang-Xin-Tuan-Hei-Gu’ (‘LTH,’ cold tolerant genotype) – and 36 comparisons with five *indica* cultivars – ‘IR24’ (photoperiod-insensitive, high yielding and cold sensitive variety), ‘IR64’ [variety with moderate tolerance toward toxicity in response to various molecules including salt, alkali, iron, and boron as well as deficiencies in phosphorus and zinc, but sensitivity to cold stress], ‘K354’ (a BC2F6 introgression line as a progeny of C418 and cold tolerant variety), ‘Huahui 1’ (’HH1,’ insect-resistant variety as a progeny of Minghui 63), and ‘Minghui 63’ (‘MH,’ heat tolerant variety and a parental line of HH1). Their upregulation was conserved between *japonica* and *indica* cultivars. All of these genes provide potential for a broader range of applications to enhance cold tolerance in rice. These 502 DEGs were used for further analysis of the cold-stress response (Supplementary Table S3).

### Validation of Cold-Inducible Genes in Rice Roots Using the *GUS* Reporter System and qRT-PCR

**FIGURE 3:**
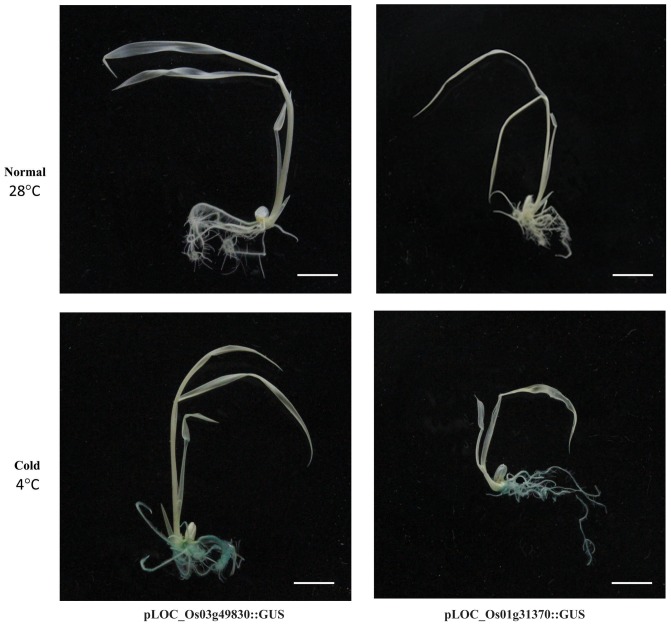
Validation of expression patterns for two cold stress-responsive genes using GUS reporter systems. Promoter trap lines using *GUS* reporter gene were selected and tested for GUS activity. Promoter trap line for *LOC_Os01g31370*, Line PFG 3A-50649 (right), and that of *LOC_Os03g49830*, Line PFG 1*C*-08613 (left) were confirmed through co-segregation test of *GUS* expression and T-DNA insertion through genotyping analysi*s.* Upper panel, GUS-staining data from promoter trap lines under normal growing conditions; lower panel, lines under cold-stress conditions. Homozygous progeny of T-DNA insertion for each of two lines were used.

Promoter traps employing the *GUS* reporter gene system have been used to identify promoters involved in regulating tissue-specific and stress-responsive expression patterns (Jung et al., 2005, 2006). Our meta-expression analysis identified the top 50 genes showing >3.5-fold upregulation by cold stress when compared with the control (Supplementary Table S3). We then searched and identified 52 potential promoter trap lines of 43 candidate genes and examined *GUS* expression patterns in 7-day-old seedlings. The lines for two genes (*PFG 3A-50649* for *LOC_Os01g31370* and *PFG 1C-08613* for *LOC_Os03g49830*) displayed *GUS* expression in the roots after plants were exposed to stress for 24 h (**Figure 3** and Supplementary Figure S1). This cold-related expression was also verified by qRT-PCR (**Supplementary Figure S1**). Previous studies using a promoter-*GUS* vector or promoter trap system have confirmed the upregulation of *LOC_Os10g41200* in response to cold stress (Su et al., 2010; Jeong and Jung, 2015). Our findings demonstrated that the promoter trap system, when combined with qualified genome-wide transcriptome data, is a very effective way for quickly identifying the activity of an endogenous promoter. This enables researchers to develop novel promoters for future applications.

### Analysis of *Cis*-Regulatory Elements Conserved in Promoters of Three Cold-Inducible Genes Confirmed by the *GUS* Reporter System

**FIGURE 4:**
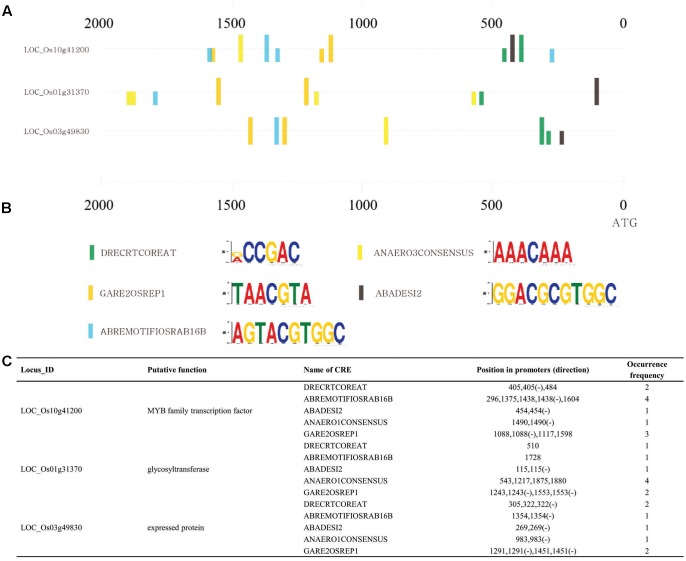
Identification of CREs conserved in three cold-inducible genes. Consensus CREs in promoters of cold-inducible genes were studied with GUS reporter systems, using 2-kb upstream sequences of ATG for *LOC_Os01g31370*, *LOC_Os03g49830*, and LOC_Os10g41200 to confirm cold induction *in planta*. **(A)** Distribution of five CREs conserved in promoters of three cold-inducible genes but not in those of randomly selected genes. **(B)** Names and conserved sequences presented using MEME suit. **(C)** Positions and frequency were determined for five CREs in promoters of above three genes.

To identify the *cis*-regulatory regulatory elements (CREs) associated with the response to cold, we used promoter regions in 2-kb sequences upstream of ATG of the two cold-inducible genes (*LOC_Os01g31370* and *LOC_Os03g49830)* that had been validated through GUS trap assays and also included the promoter region of *LOC_Os10g41200*, which have previously been reported as a cold-inducible gene using GUS reporter systems (Su et al., 2010; Rerksiri et al., 2013; Jeong and Jung, 2015). Through *in silico* analysis of CREs, we revealed the presence of common 51 CREs in the promoter regions from the PLANTPAN 2.0 database^9^ (Chow et al., 2016) and MEME tool^10^ (Bailey et al., 2006). Selected promoter regions and CREs are summarized in Supplementary Table S4. Of these, we have more interest in five unique CREs: DRECRTCOREAT (RCCGAC), ABREMOTIFIOSRAB16B (AGTACGTGGC), ABADESI2 (GGACGCGTGGC), GARE2OSREP1 (TAACGTA), and ANAERO3CONSENSUS (TCATCAC) (**Figure 4** and Supplementary Table S4). DRECRTCOREAT is a core motif of dehydration-responsive element/C-repeat (DRE/CRT) found in the promoters of genes from various species. Previous studies reported that *OsDREB1A, AtDREB1A* and *ZmDREB1A* bound to (G/ACCGAC) with the different efficiency by competitive DNA binding assays (Sakuma et al., 2002; Dubouzet et al., 2003; Qin et al., 2004) and *OsDREB* gene encodes transcription activators that function in drought, salt and cold-responsive gene expression (Dubouzet et al., 2003). However, although the Aloe DREB1 can bind to the DRE, it may also bind to other CREs effectively, which can function in a new cold-induced signal transduction pathway (Wang and He, 2007). It has been known that phytohormones including ABA, auxin, gibberellic acid (GA), salicylic acid (SA) and ethylene are related to the cold responses positively or negatively (Miura and Furumoto, 2013; Verma et al., 2016). Among the ABA-responsive CREs, we found that ABREMOTIFIOSRAB16B and ABADESI2 earlier identified from rice *Osrab16B* promoter and wheat histone H3 promoter were related to ABA-regulated transcription (Terada et al., 1993; Ono et al., 1996; Busk and Pagès, 1998). In addition, GARE1OSREP1 is involved in Gibberellin-responsive element (GARE) found in rice Os*REP-1* promoter (Ogawa et al., 2003; Sutoh and Yamauchi, 2003). ANAERO3CONSENSUS found in promoters of anaerobic genes is involved in the fermentative pathway and related to anaerobic response (Mohanty et al., 2005). In summary, DRECRTCOREAT might be related to cold-preferred expression, and ABREMOTIFIOSRAB16B, ABADESI2 and GARE1OSREP1 might be associated with crosstalk between phytohormones and cold stress-preferred expression. The other CREs not mentioned here might have novel roles in driving cold stress-preferred expression and further experiments will be required to clarify our estimation.

### Analysis of GO Enrichment Reveals Biological Processes Associated with Cold Stress Responses in Rice Roots

**FIGURE 5:**
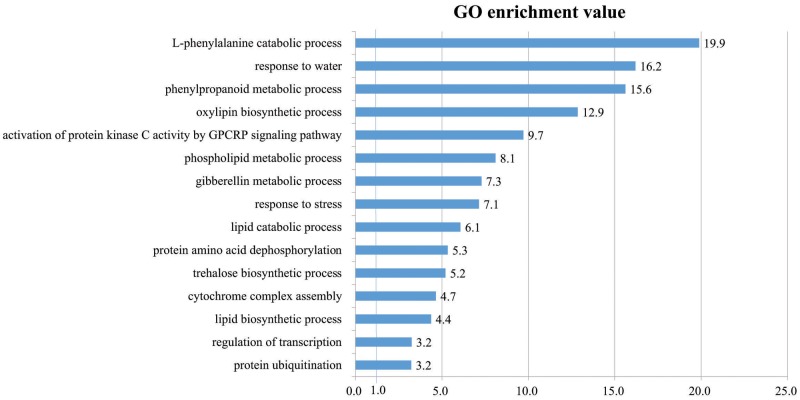
Gene Ontology enrichment analysis in ‘Biological Process’ category for genes up-regulated in response to cold stress. In all, 15 GO terms were over-represented by >2-fold enrichment value, with *p*-values < 0.05. Details of GO assignment are presented in Supplementary Table S4.

To determine the functions of 502 DEGs up-regulated by cold stress in rice roots, we studied their GO terms within the ‘biological process’ category. In all, 15 terms were highly over-represented in our gene list, with *p*-values < 0.05 and fold-enrichment values of >2-fold (**Figure 5** and Supplementary Table S5). We have also previously reported this (Jung et al., 2008b). The terms included ‘L-phenylalanine catabolic process’ (19.9-fold enrichment), ‘response to water’ (16.2), ‘phenylpropanoid metabolic process’ (15.6), ‘oxylipin biosynthetic process’ (12.9), ‘activation of protein kinase C activity by GPCRP signaling pathway’ (9.7), ‘phospholipid metabolic process’ (8.1), ‘gibberellin metabolic process’ (7.3), ‘response to stress’ (7.1), ‘lipid catabolic process ’ (6.1), ‘protein amino acid dephosphorylation’ (5.3), ‘trehalose biosynthetic process’ (5.2), ‘cytochrome complex assembly’ (4.7), ‘lipid biosynthetic process’ (4.4), ‘regulation of transcription’ (3.2), and ‘protein ubiquitination’ (3.2).

Of these, ‘L-phenylalanine catabolic process’ was the most significantly enriched by cold stress while another critical component in that response was ‘phenylpropanoid metabolic process.’ Transcriptome profile analysis of maize (*Zea mays*) seedlings in response to cold stress has shown that 31 DEGs for phenylalanine metabolism are induced (Shan et al., 2013). Transcript and metabolic profiling of *Arabidopsis thaliana* (Charlton et al., 2008) has indicated that phenylpropanoids, along with Lys, Met, Trp, Tyr, Arg, Cys, and the polyamine biosynthetic pathway, are important metabolites that are highly accumulated in response to cold stress. Profiling of maize seedling transcripts by Shan et al. (2013) has also revealed the induction of 54 DEGs for phenylpropanoid metabolism. All of these results suggest that the phenylpropanoid metabolic pathway is activated when various plant species are exposed to cold stress.

Metabolic profiling of *Camellia sinensis in* response to cold (Wang X.C. et al., 2013) has shown that expression is increased for genes involved in the signal transduction mechanism. Three oxylipin biosynthetic-related genes and two trehalose biosynthetic genes are highly expressed in cold-tolerant *Elymus nutans* (Fu et al., 2016). Moreover, transcriptomics profiling of *Lotus japonicus* under cold stress has demonstrated that those conditions lead to the upregulation of the phospholipid metabolic process (Calzadilla et al., 2016).

Transcriptome profiling has presented the upregulation of GA metabolism in cold-stressed ‘Meyer’ zoysiagrass (Wei et al., 2015) and greater than threefold induction of *gibberellin 2-beta-dioxygenase* genes in cassava, which is also related to responses to abiotic and biotic stimuli (An et al., 2012). All of these reports indicate that the gibberellin metabolic pathway is activated during periods of cold stress.

Genes for ‘lipid catabolic process,’ ‘protein amino acid dephosphorylation,’ ‘cytochrome complex assembly,’ ‘regulation of transcription,’ and ‘protein ubiquitination’ also have important roles in the abiotic-stress response (see, e.g., data in **Figure 5**). For example, in *A. thaliana*, several lipid catabolism enzymes in rice (in particular, phospholipids A and D) are activated by low temperatures, as manifested by the heightened accumulation of fatty acids (Wang et al., 2006; Usadel et al., 2008). Serine phosphorylation or dephosphorylation is involved in cold activation signaling of *Arabidopsis ICE1*, and its Ox in *Isatis tinctoria* confers cold tolerance (Chinnusamy et al., 2003; Xiang et al., 2013). Campos et al. (2003) have reported that a cold-tolerant genotype of *Coffea* sp. copes with chilling through an enhanced lipid biosynthetic process. Regulation of transcription is also important for cold tolerance. For example, in *Arabidopsis*, ICE1 and an R2R3-type MYB control the transcriptional regulation of *DREB* TFs within the mechanism for cold tolerance (Agarwal et al., 2006; Miura et al., 2007). We also identified ‘Protein ubiquitination’ as another important GO term that is also linked with cold tolerance. For example, *Arabidopsis HOS1* mediates the ubiquitination and degradation of ICE1 and negatively regulates the response to cold stress (Dong et al., 2006). In summary, the biological processes that we identified here as being closely associated with the cold-stress response provide novel and informative resources for improving our knowledge about regulatory factors involved in the molecular mechanism(s) that enable plants to cope in a low-temperature environment.

### MapMan Analysis of Cold-Related Genes in Rice Roots

The MapMan program is very effective for visualizing diverse overviews associated with high-throughput transcriptome data (Jung and An, 2012). We uploaded Locus IDs for 502 DEGs for the cold-stress response (Supplementary Table S3) to various overviews installed in that program. Among them, 79 elements were assigned to the ‘RNA’ category, 58 to ‘protein,’ 36 to ‘signaling,’ 25 to ‘miscellaneous function’ (‘misc’), 22 to ‘hormone metabolism,’ 17 to ‘stress,’ 14 to ‘development,’ 13 to ‘transport,’ 10 each to ‘lipid metabolism’ and ‘cell wall,’ 7 to ‘secondary metabolism,’ and a smaller number to other functional groups (Supplementary Table S6). Another 154 genes did not have assigned MapMan terms. In particular, the identification of 17 cold stress-regulated elements supports our proposal that they have potential significance for enhancing tolerance when our candidate genes are expressed.

### Analysis of Metabolism Overview Associated with the Cold-Stress Response in Rice

**FIGURE 6:**
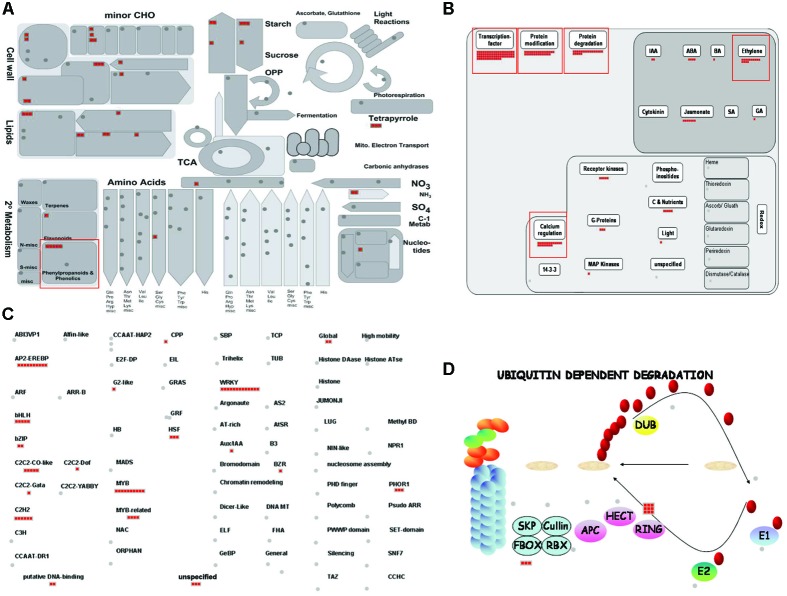
MapMan analysis of rice genes associated with response to cold stress. Overviews of Metabolism **(A)**, Regulation **(B)**, Transcription **(C)**, and Ubiquitin-mediated protein degradation pathway **(D)** were mapped with selected cold-inducible genes. Red boxes, groups of genes up-regulated by cold stress. Details are presented in Supplementary Table S6.

To investigate the significant metabolic pathways involved in the response to cold stress, we analyzed the Metabolism overview associated with 502 DEGs (**Figure 6**). Among the 44 elements found there, secondary metabolism included six for phenylpropanoids; nine for lipid metabolism, e.g., phospholipid biosynthesis and lipid degradation; 10 for cell wall metabolism, including cellulose synthase and modification; three for mitochondrial electron transport; seven for major carbohydrate (CHO) metabolism; four for minor CHO metabolism; as well as several others related to this stress, such as amino acid, nitrogen, and nucleotide metabolisms (**Figure 6A** and Supplementary Table S6). These results implied that a rice plant triggers those metabolic pathways as part of its stress response. Similar to our findings from the GO enrichment analysis, ‘L-phenylalanine catabolic process,’ ‘L-phenylalanine metabolic process,’ and category ‘secondary metabolism’ (including ‘phenylpropanoid metabolism’) were over-represented.

### Analyses of Regulation, Transcription, and Ubiquitin-Dependent Proteasome Pathway Overviews Associated with the Cold-Stress Response in Rice

Our Regulation overview of 502 DEGs demonstrated that 73 TFs, 30 genes related to protein modification, 21 associated with protein degradation, and 22 related to hormone metabolism were up-regulated in rice during periods of cold stress (**Figure 6B**). Of these, the TFs were the most abundant, meaning that they are largely involved in regulating the response and tolerance of rice to such conditions. Therefore, those genes should be considered candidates for further study to regulate the cold-stress response in rice. Accordingly, we found 13 WRKY TFs, 10 MYB and four MYB-related TFs, 10 Apetala2/Ethylene Responsive Element Binding Proteins (AP2/EREBPs), five Basic Helix-Loop-Helix (bHLH) genes, five Constans (CO)-like zinc finger family TFs, five C2H2 zinc finger family TFs, and other TFs for this response (**Figure 6C** and Supplementary Table S6).

In plants, the WRKY TFs have been more actively studied than others, and most of them have positive roles in the cold-stress response in various plant species, including *Ipomoea batatas*, where the function of a WRKY TF was first described (Ishiguro and Nakamura, 1994). This TF contains a WYRKY domain and a zinc-finger motif. Marè et al. (2004) have reported the role of *Hv-WRKY38* in the cold-stress response by *Hordeum vulgare*, and Ox of *WYRKY76* and *WYRKY71* has been shown to increase cold tolerance in rice (Yokotani et al., 2013; Kim et al., 2016). Likewise, Ox of *CsWYRKY46* in *Cucumis sativus* regulates tolerance to chilling and freezing (Zhang et al., 2016), and the cold-inducible *BcWYRKY46* from *Brassica campestris* enhances cold tolerance in transgenic tobacco (*Nicotiana tabacum*) (Wang et al., 2012). In contrast, Os*WYRKY45* and *OsWRKY13* negatively regulate cold tolerance in rice (Qiu et al., 2008; Tao et al., 2011), while *WYRKY34* mediates the cold sensitivity of mature pollen in *A. thaliana* (Zou et al., 2010) CsWRKY2, a novel *WRKY* gene from *Camellia sinensis*, is involved in cold stress responses (Wang Y. et al., 2016).

Like *WRKY* TFs, *MYB* TFs have important roles in cold tolerance. They include *OsMYB4 OsMYB2* and *MYBS3* in rice (Vannini et al., 2004; Su et al., 2010; Yang et al., 2012), *MYB15* and *HOS10* in *Arabidopsis* (Zhu et al., 2005; Agarwal et al., 2006), and *GmMYBj1* in soybean (Su et al., 2014); and *TaMYB3R1* in *Triticum aestivum* (Cai et al., 2015). Whereas all of those TFs have positive effects, *MYBC1* in *Arabidopsis* negatively regulates cold tolerance (Zhai et al., 2010).

The AP2/EREBP TFs also enhance cold tolerance. They include *JcDREB*, *JcCBF2*, *BnaERF-B3-hy15*, *DEAR1*, *ZmDREB1A*, *OsDREB1D*, and *ZmDBP4* analyzed in *Arabidopsis* (Qin et al., 2004; Tsutsui et al., 2009; Zhang et al., 2009; Wang et al., 2010, 2014; Tang et al., 2011; Xiong et al., 2013); and *JERF1*, *OsDREB1*, and *AtDREB1A* in tobacco (Kasuga et al., 2004; Li et al., 2005; Wu et al., 2007).

A major TF family of other TFs involved in cold tolerance is bHLH. *ICE1*, *ICE2*, *VabHLH1*, and *OrbHLH001* analyzed in *Arabidopsis* (Chinnusamy et al., 2003; Fursova et al., 2009; Li et al., 2010; Xu et al., 2014) and *OsbHLH1* in rice (Wang et al., 2003) are involved in cold tolerance. Next, *HOS1*, a member of the CO-like zinc finger family, regulates cold tolerance in *Arabidopsis* via *CONSTANS* degradation (Jung et al., 2012), while OsZFP245, a member of the C2H2 zinc finger family, confers such tolerance in rice (Huang et al., 2009).

Related to protein degradation, signal transduction, and hormone metabolism, a few studies have been conducted. Therefore, future analyses of uncharacterized TFs and the regulatory elements associated with protein degradation, signal transduction, and hormone metabolism identified in this study might shed the light on the effective methods for improving cold tolerance in rice.

### Evaluation of Candidate Genes Associated with Cold Stress Using Rice Genes with Known Functions

**FIGURE 7:**
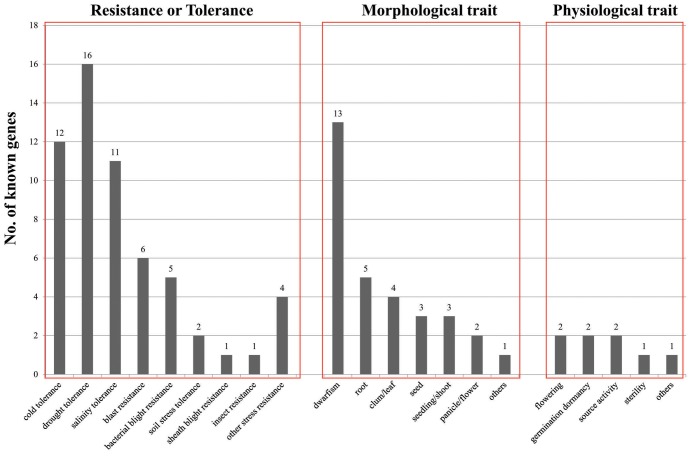
Distribution of functionally characterized genes according to three major agronomic categories. *Y*-axis, number of known genes; *X*-axis, minor functional categories in three major functional categories, presented in order of “Resistance or Tolerance,” “Morphological trait,” and “Physiological trait”.

To evaluate the significance of our candidate genes, we searched the literature to determine if functions for them have been reported previously. This was accomplished with the online OGRO database, which provides a thorough summary of rice genes that have been characterized through molecular and genetic techniques (Yamamoto et al., 2012). That summary presents the roles of 49 genes according to three agronomic trait categories: morphological, physiological, and resistance/tolerance. The functional identification of genes related to resistance/tolerance traits is the most abundant, with 27 genes being part of that category, including 12 genes involved in cold tolerance; 16, drought tolerance; 11, salinity tolerance; six, blast resistance; five, bacterial blight resistance; two, soil stress tolerance; one each for sheath blight resistance and insect resistance; and four for other stress resistances (**Figure 7**). Of these, 17 genes are partially responsible for at least two traits in that resistance/tolerance category. *OsMAPK5* and *OsWRKY45* are involved in tolerance to both biotic stress (bacterial blight and blast) and abiotic stress (drought, salinity, and cold). Others include *OsMYB2*, *ZFP182*, *OsDREB1A*, *OsDREB1B*, and *OsDREB1C*, for responses to drought, salinity, and cold; *OsbZIP52*/RISBZ5 and *OsCAF1B*, cold and drought; *OsTPP1*, cold and salinity; and *OsCPK4*, *OsCDPK7*, and *OsNAC045*, drought and salinity. The results from our transcriptome analysis had also suggested that these last three are active in the cold-stress response. We found it interesting that genes induced by low temperatures also function in other abiotic-stress responses. This implies that regulation of those responses is very complex and that intensive crosstalk might occur among them.

Regarding morphological traits, 13 genes are related to dwarfism, five to rooting, four to culms/leaves, three to seeds, three to shoots/seedlings, two to panicles/flowers, and three to other plant components (**Figure 7**). These results indicate that the cold stress-responsive genes studied here might also affect various traits, e.g., dwarfism, that can inhibit or delay normal growth. Regarding physiological traits, we found that two genes each are related to flowering, germination dormancy, and source activity, while one is related to sterility, and one to other traits (**Figure 7**). Because our findings demonstrate an interaction between cold stress and diverse morphological/physiological traits, we suggest that future studies should screen mutants and focus on their morphological and physiological phenotypes while also screening phenotypes under cold-stress conditions.

### Evaluating the Functional Significance of Cold-Inducible Genes Using a Gain-of-Function Mutant for *OsWRKY71*

**FIGURE 8:**
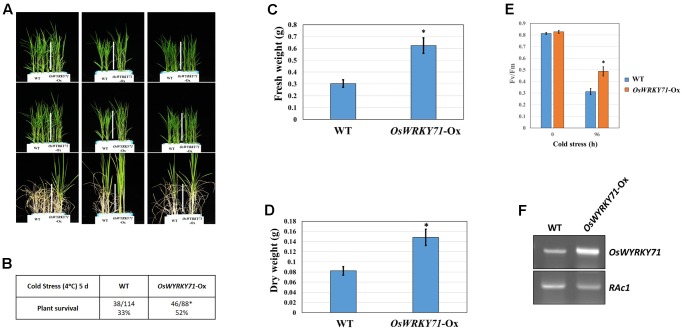
Cold-stress response mediated by *OsWYRKY71* using Ox line. **(A)** Phenotype of response by *OsWYRKY71*-Ox line observed after 10-DAG rice seedlings were exposed to cold treatment for 5 days, followed by 6 days of recovery. **(B)** Cold tolerance of *OsWYRKY71*-Ox line, based on survival rates. **(C)** Fresh weights of *OsWYRKY71*-Ox line compared with WT after recovery. **(D)** Dry weights of *OsWYRKY71*-Ox line compared with WT after recovery. **(E)** *F*_v_/*F*_m_ rates compared between *OsWYRKY71*-Ox line and WT during cold-stress period. **(F)** RT-PCR results for *OsWYRKY71*-Ox line and we used *RAc1* as an internal control.^∗^, 0.01 < *p*-value < 0.05.

Among the cold-inducible genes identified in our study,*OsWRKY71* is induced by cold stress(**Figure 1F**). As we have reported previously(Kim et al., 2016), its Ox leads to cold tolerance(**Figure 8**). The survival rate is 19% higherfor *OsWRKY71*-Ox lines than for the WT, and the transgenics also have 30% higher FWs and 60% higher DWs. Estimating*F*_v_/*F*_m_ values is a good way to depict photosynthetic efficiency under cold stress. Our data indicated that, after 96 h of chilling treatment, this efficiency in *OsWRKY71*-Ox lines decreased from 0.8 to 0.5 while that value in the WT declined from 0.8 to 0.3. Therefore, the Ox lines are 25% more efficient and *OsWRKY71* confers cold tolerance.

### Hypothetical Model for Regulating the Cold-Stress Response that Is Conserved between *japonica* and *indica* Rice Cultivars

**FIGURE 9:**
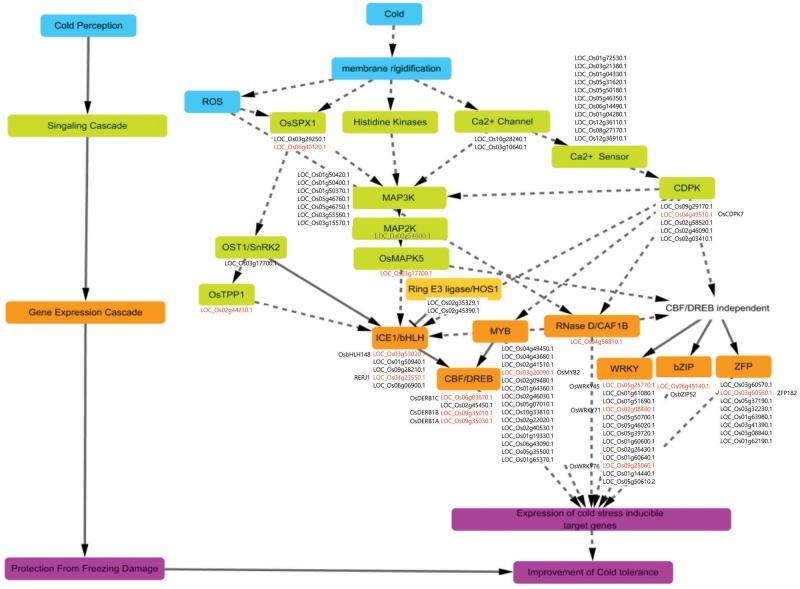
Overview of regulatory pathway for cold-stress signaling in rice. Signaling and transcriptional regulatory pathways for cold tolerance have four steps: cold perception (blue boxes), signaling cascades (green boxes), gene expression cascades (brown boxes)/protein degradation (light-brown boxes), and protection from cold (tolerance) through activation of target genes (pink boxes). Cold-inducible candidate genes were mapped to individual boxes and are presented as locus IDs. Red-colored locus IDs are genes previously characterized for cold-stress responses, with each name indicated either on left side or below corresponding ID. Brown-colored locus IDs are genes previously characterized but not directly linked with cold-stress response.

The response to low temperatures can be divided into four steps: perception of cold stress, signaling cascades for the response, regulation of gene expression, and protection from freezing damage. Our proposed model (**Figure 9**) is based on published physiological and biochemical aspects as well as reports of functions for genes involved in the relevant signaling and transcriptional pathways.

We theorize that the first reaction by a plant to chilling is to increase membrane rigidity. This is followed by the generation of ROS, then regulation of phosphate homeostasis and activation of calcium receptors and histidine kinases. The ensuing signal transduction cascades are coupled with signal perception. Examples include MAP kinase cascades and a two-component signaling system by histidine kinase. The former is more likely because the cascades of MAP kinase (OsMAPK5/LOC_Os03g17700.1), MAP kinase kinase (MAP2K; OsMKK4/LOC_Os02g54600.1), and MAP kinase kinase kinase (MAP3K, seven members in **Figure 9**), are stimulated in response to cold stress, making them the most probable candidates for this pathway. Of them, it has been known that *OsMAPK5* positively regulates tolerance to cold temperatures and other sources of stress (Xiong and Yang, 2003).

For the latter possibility, the processes might be more complex. In response to cold, plants use Ca^2+^ as a signal. Although we did not yet identify the histidine kinase genes in rice showing significant induction under cold stress, the signal received by a Ca^2+^ channel might bind to a Ca^2+^ sensor, such as calmodulin (CaM), and CaM-like protein might stimulate Ca^2+^/CaM-dependent protein kinases as suggested in **Figure 9**. Thereafter, gene expression is regulated by TFs through a process that incorporates CBF/DREB-dependent or -independent pathways.

In the case of the CBF/DREB-dependent pathway, a signal from the map kinase cascades is recognized by ICE1, which encodes a bHLH TF that activates the expression of DREB genes in the downstream pathway by directly binding the promoter regions. This results in stimulation of cold stress-responsive genes that are required for altering cellular metabolism. OsbHLH148 or RERJ1 are probable candidate genes, having the same roles as ICE1 in *Arabidopsis*, i.e., OsbHLH148 is involved in drought tolerance and RERJ1 functions in normal plant growth and development (Seo et al., 2011). OsDREB1A, OsDREB1B, and OsDREB1C have roles in tolerance to cold, drought, and salinity by triggering the expression of target genes (Ito et al., 2006). Regarding the CRT/DREB-independent pathway, TFs such as OsWRKY71 (Kim et al., 2016), OsWRKY76 (Yokotani et al., 2013), OsbZIP52 (Liu et al., 2012), ZFP182 (Huang et al., 2012), and OsMYB2 (Yang et al., 2012) are components of the trait for cold stress response. For example, a rice line that over-expressor of OsbZIP52 displays a cold-sensitive phenotype (Liu et al., 2012) and the application of such stress induces the expression of OsbZIP52, which then negatively affects the extent of that tolerance.

Although the functions of most genes for cold tolerance have not yet been defined, other types of TFs identified in our meta-expression and MapMan analyses might also be important for regulating tolerance, as indicated by the TF overview presented by MapMan (**Figure 6** and Supplementary Table S6).

Among other processes, HOS1, encoding the ring type E3 ligase, participates in the degradation process of ICE1 that is stimulated at low temperature, resulting in inactivation of the CRT/DREB-dependent transcription regulation pathway (Chinnusamy et al., 2003; Dong et al., 2006). Likewise, OST1, encoding the well-known Ser /Thr protein kinase, is activated in response to cold and phosphorylates ICE1, leading to its stability and transcriptional activity (Ding et al., 2015). However, OST1 also hinders the interaction between HOS1 and ICE1, subsequently leading to the degradation of ICE1 under cold stress when HOS1 is suppressed. OsCAF1B, with RNase D activity, functions in post-transcriptional regulation and may affect various pathways for cold tolerance (Chou et al., 2014). OsTPP1 has a role in resistance to abiotic stress. At low temperatures, it also positively regulates the expression of tolerance genes by participating in the glucose deprivation signaling pathway (Ge et al., 2008). Despite these numerous reports, however, all of these hypotheses must still be verified through further experiments.

Cold stress is one of the main environmental factors that adversely affect plant growth and yield. Thus, it is important that we understand this stress signaling and its regulatory network if we are to develop cultivars with greater tolerance. To this end, we have produced a hypothetical model that considers our current findings as well as data derived from earlier research.

## Conclusion

Our study goal was to identify low-temperature-responsive genes that can be commonly used by rice researchers throughout the world. For this, we collected a broad range of genome-wide transcriptome data produced from plants under low-temperature conditions. This information included data deposited from published microarrays or re-processed from RNA-seq analyses. The 502 genes identified here are conserved between *japonica* and *indica* cultivars, two representative subspecies of rice. Results of bioinformatics analyses using GO enrichment and MapMan tools for these candidate genes was applied to reveal important biological processes and related metabolic and regulatory pathways. In addition, we constructed a possible regulatory network based on such information. Serving as a valuable foundation for future research, our proposed model can help in the discovery of key regulatory genes that confer cold tolerance. This can be accomplished by using a gene-indexed mutant collection or biotechnological approaches that are well-established in rice.

## Author Contributions

K-HJ, MK, and S-RK design overall experimental schemes. MK and Y-SG performed experiments. MK and K-HJ wrote manuscript.

## Conflict of Interest Statement

The authors declare that the research was conducted in the absence of any commercial or financial relationships that could be construed as a potential conflict of interest.

## Footnotes

1 http://qtaro.abr.affrc.go.jp/ogro

2 http://mapman.gabipd.org

3 http://www.generunner.net/

4 http://www.ncbi.nlm.nih.gov/tools/primer-blast/

5 http://www.ncbi.nlm.nih.gov/geo/

6 http://plantpan2.itps.ncku.edu.tw/

7 http://www.nbcr.net/

8 http://www.ricearray.org/analysis/go_enrichment.shtml

9 http://plantpan2.itps.ncku.edu.tw

10 http://meme-suite.org/

## Supplementary Material

FIGURE S1 Validation of expression patterns for three genes (LOC_Os03g49830, LOC_Os10g41200, and LOC_Os01g31370) under cold stress using qRT-PCR analysis. ^∗∗∗^, *p*-value < 0.001.

## Abbreviations

CREs: *cis*-regulatory elements
DAG: days after germination
DEG: differentially expressed gene
FASTA: Fast Alignment Search Tool
GEO: gene expression omnibus
GO: gene ontology
GUS: ββ-glucuronidase
KMC: K-means clustering
MAST: Motif Alignment and Search Tool
Mev: Multiple Experiment Viewer
MS: Murashige and Skoog
NCBI: National Center for Biotechnology Information
OGRO: overview of functionally characterized genes in rice online database
Ox: overexpression
PPI: protein–protein interaction
RGAP: Rice Genome Annotation Project
TF: transcription factor
TOMTOM: TF-binding site motifs found by the motif comparison tool
WT: wild type.
