# Supplementary figures and images for: Genome-Wide Identification and Analysis of Genes, Conserved between japonica and indica Rice Cultivars, that Respond to Low-Temperature Stress at the Vegetative Growth Stage
Source: Front Plant Sci. 2017 Jun 30;8:1120. doi: 10.3389/fpls.2017.01120 (PMC5491850; doi:10.3389/fpls.2017.01120)

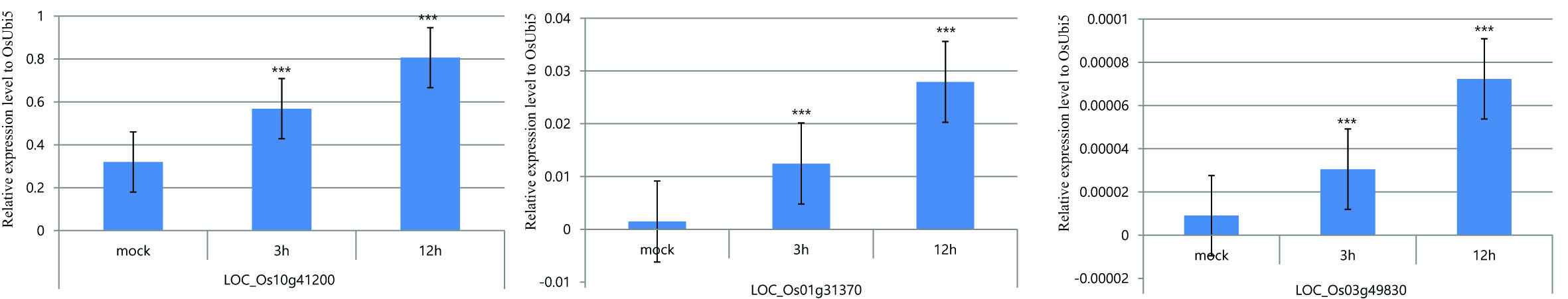

Supplement: FIGURE S1 — Validation of expression patterns for three genes (LOC_Os03g49830, LOC_Os10g41200, and LOC_Os01g31370) under cold stress using qRT-PCR analysis. ∗∗∗, p-value < 0.001. [file Image_1.JPEG]
