# Supplementary material for: Genome-Wide Identification and Analysis of Genes, Conserved between japonica and indica Rice Cultivars, that Respond to Low-Temperature Stress at the Vegetative Growth Stage
Source: Front Plant Sci. 2017 Jun 30;8:1120. doi: 10.3389/fpls.2017.01120 (PMC5491850; doi:10.3389/fpls.2017.01120)
Supplement: Supplementary file 2 [file Table_1.DOCX]

**Table S1. List of primers used for PCR analysis in this study.**

| Primer Name | Sequence (5’-3’) | Purpose |
| --- | --- | --- |
| OsZFP182-F | GACAAGGAGCACAGGGACAA | Cold-inducible marker |
| OsZFP182-R | CTAGGCGAACAACTCCAGCA | Cold-inducible marker |
| OsWRKY71-F | AGAACAGCGACGGCTCCGGCAAG | Cold-inducible marker |
| OsWRKY71-R | GATCGATCGAACTCCGCCATGG | Cold-inducible marker |
| Os01g31370-F | AACCGTACCATCCAGGACTT | Cold-inducible promoter gene |
| Os01g31370-R | TTCCGTGACACGATCACCAG | Cold-inducible promoter gene |
| Os03g49830-F | TCTGCACCGAGAGCCTCGGC | Cold-inducible promoter gene |
| Os03g49830-R | CGAGCAGTGGTAGTTCACCG | Cold-inducible promoter gene |
| Os10g41200-F | ATTCTCGGTTCCTTTCTGGC | Cold-inducible promoter gene |
| Os10g41200-R | GGACTCTCCTATGCTGAGCT | Cold-inducible promoter gene |
| OsUbi5-F | TGACGGGGAAGACGATCACGCT | Internal control |
| OsUbi5-R | GAGCCTACGCCTAAGCCTGCTG | Internal control |
| *RAc1-F* | CATGCTATCCCTCGTCTCGACCT | Internal control |
| *RAc1-R* | CGCACTTCATGATGGAGTTGTAT | Internal control |
