# Supplementary material for: Genome-Wide Identification and Analysis of Genes, Conserved between japonica and indica Rice Cultivars, that Respond to Low-Temperature Stress at the Vegetative Growth Stage
Source: Front Plant Sci. 2017 Jun 30;8:1120. doi: 10.3389/fpls.2017.01120 (PMC5491850; doi:10.3389/fpls.2017.01120)
Supplement: Supplementary file 3 [file Table_2.DOCX]

| **Table S2. Details for stress-responsive meta-expression microarray samples.** | | |
| --- | --- | --- |
| **DataSet-ID** | **Treatment** | **Variety** |
| GSE26280 | Drought-roots at tillering stage_1 | IR64 (drought-sensitive) |
| GSE26280 | Drought-roots at tillering stage_2 | IR64 |
| GSE26280 | Drought-roots at tillering stage_3 | IR64 |
| GSE26280 | Drought-roots at panicle elongation stage_1 | IR64 |
| GSE26280 | Drought-roots at panicle elongation stage_2 | IR64 |
| GSE26280 | Drought-roots at panicle elongation stage_3 | IR64 |
| E-MEXP-2401 | Drought-seedling_1 | IR64 |
| E-MEXP-2401 | Drought-seedling_2 | IR64 |
| E-MEXP-2401 | Drought-seedling_3 | IR64 |
| E-MEXP-2401 | Drought-seedling_1 | N22 (drought-resistant) |
| E-MEXP-2401 | Drought-seedling_2 | N22 |
| E-MEXP-2401 | Drought-seedling_3 | N22 |
| GSE24048 | Drought-leaf_1 | Bala (drought-sensitive) |
| GSE24048 | Drought-leaf_2 | Bala |
| GSE24048 | Drought-leaf_3 | Bala |
| GSE26280 | Drought-leaf at tillering stage_1 | IR64 |
| GSE26280 | Drought-leaf at tillering stage_2 | IR64 |
| GSE26280 | Drought-leaf at tillering stage_3 | IR64 |
| GSE26280 | Drought-leaf at panicle elongation stage_1 | IR64 |
| GSE26280 | Drought-leaf at panicle elongation stage_2 | IR64 |
| GSE26280 | Drought-leaf at panicle elongation stage_3 | IR64 |
| GSE26280 | Drought-leaf at booting stage_1 | IR64 |
| GSE26280 | Drought-leaf at booting stage_2 | IR64 |
| GSE26280 | Drought-leaf at booting stage_3 | IR64 |
| GSE21651 | Drought-leaf_1 | Drought-sensitive^a^ |
| GSE21651 | Drought-leaf_2 | Drought-sensitive^a^ |
| GSE21651 | Drought-leaf_1 | Drought-tolerant^a^ |
| GSE21651 | Drought-leaf_2 | Drought-tolerant^a^ |
| GSE24048 | Drought-leaf_1 | Azucena (drought-resistant) |
| GSE24048 | Drought-leaf_2 | Azucena |
| GSE24048 | Drought-leaf_3 | Azucena |
| GSE25176 | Drought(D1)-flag leaf_1 | IRAT109 (drought-resistant) |
| GSE25176 | Drought(D1)-flag leaf_2 | IRAT109 |
| GSE25176 | Drought(D2)-flag leaf_1 | IRAT109 |
| GSE25176 | Drought(D2)-flag leaf_2 | IRAT109 |
| GSE25176 | Drought(D3)-flag leaf_1 | IRAT109 |
| GSE25176 | Drought(D3)-flag leaf_2 | IRAT109 |
| GSE26280 | Drought-panicle_1 | IR64 |
| GSE26280 | Drought-panicle_2 | IR64 |
| GSE26280 | Drought-panicle_3 | IR64 |
| GSE6901 | Drought_seedling_1 | IR64 |
| GSE6901 | Drought_seedling_2 | IR64 |
| GSE6901 | Drought_seedling_3 | IR64 |
| GSE25176 | Drought(D1)-flag leaf_1 | ZS97 (drought-sensitive) |
| GSE25176 | Drought(D1)-flag leaf_2 | ZS97 |
| GSE25176 | Drought(D2)-flag leaf_1 | ZS97 |
| GSE25176 | Drought(D2)-flag leaf_2 | ZS97 |
| GSE25176 | Drought(D3)-flag leaf_1 | ZS97 |
| GSE25176 | Drought(D3)-flag leaf_2 | ZS97 |
| GSE6901 | Salt-seedling_1 | IR64 |
| GSE6901 | Salt-seedling_2 | IR64 |
| GSE6901 | Salt-seedling_3 | IR64 |
| GSE16108 | Salt-seedling_1 | Bulk sensitive |
| GSE16108 | Salt-seedling_2 | Bulk sensitive |
| GSE16108 | Salt-seedling_1 | Bulk tolerant |
| GSE16108 | Salt-seedling_2 | Bulk tolerant |
| GSE16108 | Salt-seedling_1 | CSR27 (salt-tolerant) |
| GSE16108 | Salt-seedling_2 | CSR27 |
| GSE16108 | Salt-seedling_1 | MI48 (salt-sensitive) |
| GSE16108 | Salt-seedling_2 | MI48 |
| GSE21651 | Salt-leaf_1 | Salt-sensitive^a^ |
| GSE21651 | Salt-leaf_2 | Salt-sensitive^a^ |
| GSE21651 | Salt-leaf_1 | Salt-tolerant^a^ |
| GSE21651 | Salt-leaf_2 | Salt-tolerant^a^ |
| GSE6901 | Cold-seedling_1 | IR64 |
| GSE6901 | Cold-seedling_2 | IR64 |
| GSE6901 | Cold-seedling_3 | IR64 |
| GSE33204 | Cold (12℃)-leaf_1 | Huahui 1 |
| GSE33204 | Cold (12℃)-leaf_2 | Huahui 1 |
| GSE33204 | Cold (12℃)-leaf_3 | Huahui 1 |
| GSE33204 | Cold (12℃)-leaf_1 | Minghui 63 |
| GSE33204 | Cold (12℃)-leaf_2 | Minghui 63 |
| GSE33204 | Cold (12℃)-leaf_3 | Minghui 63 |
| GSE37940 | Cold (4℃, 2 h)-leaf_1 | K354, a BC2F6 introgression line with cold tolerance |
| GSE37940 | Cold (4℃, 2 h)-leaf_2 | K354 |
| GSE37940 | Cold (4℃, 2 h)-leaf_3 | K354 |
| GSE37940 | Cold (4℃, 24 h)-leaf_1 | K354 |
| GSE37940 | Cold (4℃, 24 h)-leaf_2 | K354 |
| GSE37940 | Cold (4℃, 24 h)-leaf_3 | K354 |
| GSE37940 | Cold (4℃, 12 h)-leaf_1 | K354 |
| GSE37940 | Cold (4℃, 12 h)-leaf_2 | K354 |
| GSE37940 | Cold (4℃, 12 h)-leaf_3 | K354 |
| GSE37940 | Cold (4℃, 48 h)-leaf_1 | K354 |
| GSE37940 | Cold (4℃, 48 h)-leaf_2 | K354 |
| GSE37940 | Cold (4℃, 48 h)-leaf_-3 | K354 |
| GSE37940 | Cold (4℃, 6 h)-leaf_1 | K354 |
| GSE37940 | Cold (4℃, 6 h)-leaf_2 | K354 |
| GSE37940 | Cold (4℃, 6 h)-leaf_3 | K354 |
| GSE38023 | Cold (2 h)-leaf_1 | IR29 (chilling-sensitive) |
| GSE38023 | Cold (2 h)-leaf_2 | IR29 |
| GSE38023 | Cold (2 h)-leaf_3 | IR29 |
| GSE38023 | Cold (8 h)-leaf_1 | IR29 |
| GSE38023 | Cold (8 h)-leaf_2 | IR29 |
| GSE38023 | Cold (8 h)-leaf_3 | IR29 |
| GSE38023 | Cold (24 h)-leaf_1 | IR29 |
| GSE38023 | Cold (24 h)-leaf_2 | IR29 |
| GSE38023 | Cold (24 h)-leaf_3 | IR29 |
| GSE38023 | Cold (48 h)-leaf_1 | IR29 |
| GSE38023 | Cold (48 h)-leaf_2 | IR29 |
| GSE38023 | Cold (48 h)-leaf_3 | IR29 |
| GSE38023 | Cold (recover 24 h)-leaf_1 | IR29 |
| GSE38023 | Cold (recover 24 h)-leaf_2 | IR29 |
| GSE38023 | Cold (recover 24 h)-leaf_3 | IR29 |
| GSE37940 | Cold (4℃, 2 h)-shoot_1 | C418, a japonica restorer line |
| GSE37940 | Cold (4℃, 2 h)-shoot_2 | C418 |
| GSE37940 | Cold (4℃, 2 h)-shoot_3 | C418 |
| GSE37940 | Cold (4℃, 24 h)-shoot_1 | C418 |
| GSE37940 | Cold (4℃, 24 h)-shoot_2 | C418 |
| GSE37940 | Cold (4℃, 24 h)-shoot_3 | C418 |
| GSE37940 | Cold (4℃, 12 h)-shoot_1 | C418 |
| GSE37940 | Cold (4℃, 12 h)-shoot_2 | C418 |
| GSE37940 | Cold (4℃, 12 h)-shoot_3 | C418 |
| GSE37940 | Cold (4℃, 48 h)-shoot_1 | C418 |
| GSE37940 | Cold (4℃, 48 h)-shoot_2 | C418 |
| GSE37940 | Cold (4℃, 48 h)-shoot_3 | C418 |
| GSE37940 | Cold (4℃, 6 h)-shoot_1 | C418 |
| GSE37940 | Cold (4℃, 6 h)-shoot_2 | C418 |
| GSE37940 | Cold (4℃, 6 h)-shoot_3 | C418 |
| GSE38023 | Cold (2 h)-leaf_1 | Li-Jiang-Xin-Tuan-Hei-Gu (chilling-tolerant) |
| GSE38023 | Cold (2 h)-leaf_2 | Li-Jiang-Xin-Tuan-Hei-Gu |
| GSE38023 | Cold (2 h)-leaf_3 | Li-Jiang-Xin-Tuan-Hei-Gu |
| GSE38023 | Cold (8 h)-leaf_1 | Li-Jiang-Xin-Tuan-Hei-Gu |
| GSE38023 | Cold (8 h)-leaf_2 | Li-Jiang-Xin-Tuan-Hei-Gu |
| GSE38023 | Cold (8 h)-leaf_3 | Li-Jiang-Xin-Tuan-Hei-Gu |
| GSE38023 | Cold (24 h)-leaf_1 | Li-Jiang-Xin-Tuan-Hei-Gu |
| GSE38023 | Cold (24 h)-leaf_2 | Li-Jiang-Xin-Tuan-Hei-Gu |
| GSE38023 | Cold (24 h)-leaf_3 | Li-Jiang-Xin-Tuan-Hei-Gu |
| GSE38023 | Cold (48 h)-leaf_1 | Li-Jiang-Xin-Tuan-Hei-Gu |
| GSE38023 | Cold (48 h)-leaf_2 | Li-Jiang-Xin-Tuan-Hei-Gu |
| GSE38023 | Cold (48 h)-leaf_3 | Li-Jiang-Xin-Tuan-Hei-Gu |
| GSE38023 | Cold (recover 24 h)-leaf_1 | Li-Jiang-Xin-Tuan-Hei-Gu |
| GSE38023 | Cold (recover 24 h)-leaf_2 | Li-Jiang-Xin-Tuan-Hei-Gu |
| GSE38023 | Cold (recover 24 h)-leaf_3 | Li-Jiang-Xin-Tuan-Hei-Gu |
| GSE33204 | Heat (45℃)-leaf_1 | Huahui 1 |
| GSE33204 | Heat (45℃)-leaf_2 | Huahui 1 |
| GSE33204 | Heat (45℃)-leaf_3 | Huahui 1 |
| GSE33204 | Heat (45℃)-leaf_1 | Minghui 63 |
| GSE33204 | Heat (45℃)-leaf_2 | Minghui 63 |
| GSE33204 | Heat (45℃)-leaf_3 | Minghui 63 |
| GSE18930 | Submergence for 1 day | M202 |
| GSE18930 | Submergence for 1 day | M202 |
| GSE18930 | Submergence for 1 day | M202(SUB1) |
| GSE18930 | Submergence for 1 day | M202(SUB1) |

^a^ Names of variety were not specified from NCBI GEO.
